# Meta-analysis of the association between the inflammatory potential of diet and urologic cancer risk

**DOI:** 10.1371/journal.pone.0204845

**Published:** 2018-10-01

**Authors:** Dong-Liang Lu, Zheng-Ju Ren, Qin Zhang, Peng-Wei Ren, Bo Yang, Liang-Ren Liu, Qiang Dong

**Affiliations:** 1 Department of Urology, Institute of Urology, West China Hospital, Sichuan University, Chengdu, Sichuan, China; 2 Department of Radiology, Chongqing Traditional Chinese Medicine Hospital, Chongqing, China; 3 Department of Evidence-Based Medicine and Clinical Epidemiology, West China Hospital, Sichuan University, Chengdu, Sichuan, China; University of Ioannina Medical School, GREECE

## Abstract

**Background:**

The inflammatory potential of diet has been shown to have an association with the risk of several cancer types, but the evidence is inconsistent regarding the related risk of urologic cancer (UC). Therefore, we conducted the present meta-analysis to investigate the association between the inflammatory potential of diet and UC.

**Methods:**

PubMed, Embase and Web of Science were searched up to July 31, 2018. Two reviewers independently selected the studies and extracted the data. The pooled risk ratio (RR) and its 95% confidence interval (CI) were calculated using the Stata12.0 software package.

**Results:**

Nine case-control studies and three cohort studies including 83,197 subjects met the inclusion criteria. The overall meta-analysis results showed that individuals with the highest category of DII (dietary inflammatory index) were associated with an increased risk of prostate cancer (RR = 1.62, 95% CI: 1.30–2.02); subgroup analysis showed consistent results. For kidney and bladder cancer, significant positive associations were found in individuals with the highest category of DII score; however, no significant association was found between DII and the risk of urothelial cell carcinoma (UCC).

**Conclusion:**

Available data suggest that more pro-inflammatory diets are associated with an increased risk of prostate cancer, kidney cancer and bladder cancer. However, further well designed large-scaled cohort studies are warranted to provide more conclusive evidence.

## Introduction

Prostate cancer, bladder cancer, and kidney cancer, the most common urologic tumors, are leading causes of cancer-related morbidity and mortality worldwide [1, 2]. Despite rapid advances in early diagnosis and therapy over the past few decades, the incidence and mortality rates of urologic cancer continue to increase [1–3]. In 2017, approximately 146,650 new urologic cancer cases and 32,190 deaths were projected to occur in the United States [4]. The etiology of urologic cancer is complicated and not yet fully elucidated. Considerable evidence indicates that chronic inflammation plays a key role in carcinogenesis; several studies have supported the involvement of upregulated pro-inflammatory molecules in tumor progression [5–7]. Diet is a major source of bioactive compounds that can be grouped into pro-inflammatory and anti-inflammatory components [8]. A diet rich in fruits, vegetables, healthy oils, and fish may have been associated with lower levels of inflammation and with decreased cancer risk [9, 10]. In contrast, high intakes of PUFA, mainly n-6 fatty acids, are associated with higher levels of inflammation and an increased risk of cancer [11, 12]. Therefore, adopting an anti-inflammatory diet may reduce UC risk.

The DII score, a literature-derived population-based dietary score, was developed to estimate the inflammatory potential of nutrients and foods in the context of a dietary pattern [13, 14]. The DII score was computed from dietary intake assessed using a validated food frequency questionnaire or 24-h recall dietary records [13]. Individuals’ intakes from these diverse populations could be expressed to the range of intakes of forty-five food parameters according to food consumption data sets from countries around the world, and DII scores were multiplied by individuals’ intakes of food parameters [14]. The pro-inflammatory diet was associated with a higher DII score and anti-inflammatory diet was associated with a lower DII score [13–15]. Diet and nutrients are modifiable factors which may influence carcinogenesis of urinary tract, however, there was no specific diet been reported to prevent urologic carcinogenesis [16]. Recently, several large-scale prospective cohort and case-control studies were published. These studies reported the association between the inflammatory potential of diet and the risk of UC; the results from these studies remain controversial. Graffouillère et al. reported that pro-inflammatory diets are associated with increased prostate cancer risk in French middle-aged adults [17]. However, such a significant association was not detected in other studies [18, 19]. Vázquez-Salas et al. reported that a pro-inflammatory diet is not related to prostate cancer risk or prostate cancer aggressiveness [19]. In addition, the inflammatory potential of diet may influence the prognosis of patients with more aggressive prostate cancer [20]. It is thus critical to synthesize available evidence on the potential relation between a pro-inflammatory diet and UC risk since, and to our knowledge, this is the first meta-analysis to examine the association between the inflammatory potential of diet and UC risk.

## Methods

### Search strategy

The included studies were searched from PubMed, Embase and Web of Science up to July 31, 2018. Search terms were as follows: “(inflammatory potential of diet OR dietary inflammatory index OR pro-inflammatory diet OR anti-inflammatory diet) AND (urologic OR urinary tract OR prostate OR renal OR kidney OR bladder) AND (cancer OR carcinoma OR neoplasm).” In addition, a manual search of references in relevant articles was conducted to find other eligible studies. The search strategy flowchart is shown in S1 Fig.

### Selection criteria

Only studies meeting the following inclusion criteria were eligible: 1) studies with full text articles; 2) studies that reported the association between the inflammatory potential of diets and UC risk; 3) studies with odds ratios (ORs), relative risks (RRs), or hazard ratios (HRs) with corresponding 95% confidence intervals (CIs); and 4) the published language was English. Exclusion criteria were as follows: (1) studies referred to outcomes other than UC; (2) studies with partially unusable data; (3) review articles, meta-analyses, animal studies, conference abstracts or editorial articles.

### Quality assessment

A quality assessment of the included studies was evaluated by the Newcastle-Ottawa Scale (NOS) [21]. The NOS, categorized into three aspects—selection, comparability, and exposure—was composed of eight items for both case–control and cohort studies. The methodological quality of studies is judged using a ‘‘star” rating system (maximum nine stars). Scores range from 0 stars (worst) to 9 stars (best), and studies with a score ≥7 were defined as high quality. Discrepancies in opinions were resolved by discussion and consensus.

### Data extraction

Data extraction was independently conducted by two authors using a collection form that was checked by a third author. Disagreement was resolved through discussion and consensus finding. For each study, we collected the following information: (1) the first author’s name, year of publication, country, ethnicity, and sample size of the study; (2) mean age or age range, study design, and cancer type; (3) dietary assessment method, most fully adjusted risk estimate, and cofounders included in the final models.

### Statistical analysis

The multivariable-adjusted HR or OR with 95% CI for the highest versus the lowest DII score were pooled using random effects models. A Chi-square-based Q test and the I^2^ metric were used to assess heterogeneity among studies. The heterogeneity was considered significant when p<0.10 and I^2^>50%. Given that the included studies were conducted at a global level and addressed different types of cancer, random effects model was used to get more conservative results. Subgroup analyses based on study design and sample size were performed for prostate cancer. The significance of the summary OR was determined by the Z-test, and P<0.05 was considered as statistically significant. Begg’s, Egger’s test and funnel plots were used to assess potential publication bias. Sensitivity analysis was performed by excluding one study each time to evaluate the stability of the results. Statistical analysis was performed using STATA software version 12.0 (Stata Corporation, College Station, Texas, USA).

## Results

### Study characteristics

A total of 855 results were retrieved through literature searching. Of these, 843 studies were excluded based on inclusion and exclusion criteria. Finally, twelve studies considering 83,197 subjects met the inclusion criteria [17–19, 22–30]. Four studies were conducted in Europe, 5 in America, 2 in Asia, and 1 in Australia. Moreover, eight studies reported the relationship between a pro-inflammation diet and the incidence of prostate cancer; additionally, two studies for kidney cancer, one study for bladder cancer and one study for urothelial cell carcinoma described the relationship between cancer incidence and a pro-inflammation diet. The articles were published between 2015 and 2018. The median follow-up time of cohort studies was 6.33 years (range 4–11). Detailed characteristics of all included studies are shown in Table 1. Study quality was evaluated by using the NOS; studies with scores ≥7 were considered to have high quality. Two studies had a score of 8, 9 studies had a score of 7, and 1 study had a score of 6. Study quality based on the NOS score is presented in Table 2.

**Table 1:** Characteristics of studies included in meta-analysis.

Study/Year	Country	Sample size	Source of control	Mean age or range (years)	Study design	Cancer type	Dietary assessment	Number of food parameters	Mean DII value (SD or range)	HR or OR (highest vs. lowest) (95% CI)	Follow-up (years)	Adjustment for covariates
Shivappa1 et al.2015 [22]	Jamaica	Case: 229 Control:250	Outpatients	Case: 67.8 Control: 62.0	Case-control	Prostate	FFQ (70 items)	21	Case and control: -1.05±1.11	2.39(1.14,5.04)	—	Age, BMI, smoking status, education, physical activity, energy intake, family history of PC
Shivappa2 et al.2017 [23]	Canada	Case:72 Control:302	Outpatients	Case: 65.1 Control: 63.5	Case-control	Prostate	FFQ (67 items)	18	NA	3.5(1.25,9.8)	—	Age, family history of PC, physical activity as a teenager, and energy intake
Shivappa3 et al.2017 [28]	Iran	Case: 50 Control:100	Hospital based	Case: 57.4 Control: 56.9	Case-control	Prostate	FFQ (168 items)	25	NA	3.96 (1.29, 12.16)	—	Age, total energy intake, BMI, smoking status, marital status and family history of cancer, diabetes, hypertension, and cardiovascular diseases
Graffouillère et al.2016 [17]	France	2771	—	49.26	Cohort	Prostate	—	36	0.3±1.8	2.08(1.06,4.09)	12.6	Age, sex, intervention group of the initial SU.VI.MAX trial, number of 24-h dietary records, BMI, height, physical activity, smoking status, educational level, energy intake without alcohol, and alcohol intake, baseline PSA and family history of PC in first-degree relatives
Vázquez-Salas et al.2016 [19]	Mexico	Case:394 Control:794	Population based	Case: 67.7 Control: 66.9	Case-control	Prostate	FFQ (127 items)	27	Case:0.43(-4.59~3.50) Control: 0.52(-4.47~4.51)	1.18(0.85,1.63)	—	Age, educational level, history of PC in first-degree relatives, BMI 2 years before the interview, physical activity throughout life, smoking status 5 years before the interview, history of chronic diseases
Shivappa4 et al.2015 [25]	Italy	Case:1294 Control:1451	Hospital based	Case: 46–74 Control: 46–74	Case-control	Prostate	FFQ (78 items)	31	NA	1.33(1.01,1.76)	—	Age, study center, BMI, years of education, social class, smoking status, family history of PC, and total energy intake.
Shivappa8 et al.2018 [29]	Iran	Case:60 Control:60	Hospital based	Case:66.0 Control:61.4	Case-control	Prostate	FFQ (160 items)	25	Case: 1.55±1.16 control: 0.93±1.4	2.60 (1.05, 6.41)	—	Age, ethnicity, BMI, education, physical activity, smoking status, and use of aspirin
Shivappa9 et al.2018 [30]	Argentina	Case:153 Control:309	Population based	Case: 48–89 Control: 46–89	Case-control	Prostate	FFQ (127 items)	22	NA	1.50 (1.24–1.80)	—	Age, usual BMI, energy intake, occupational exposure, family history of cancer
Shivappa6 et al.2017 [24]	Italy	Case: 767 Control: 1534	Hospital based	Case: 24–79 Control: 22–79	Case-control	Kidney	FFQ (78 items)	31	Case: 0.13±1.349 control: -0.06±1.38	1.41(1.02,1.97) Male:1.28 (0.85, 1.92) Female:1.68 (0.93, 3.03)	—	Conditioned on study center, sex, and quinquennia of age and adjusted for energy intake, year of interview, education, BMI, tobacco smoking, and family history of RCC
Shivappa7 et al.2017 [27]	USA	33817	—	55–69	Cohort	Kidney	FFQ (121 items)	29	Case and control: −0.87 ±2.02	Female:1.52(1.09,2.13)	—	Age, BMI, smoking77 status, pack-years of smoking, education, HRT use, hypertension, total energy intake
Dugué et al.2016 [18]	Australia	37442	—	27–76	Cohort	UCC	FFQ (121 items)	29	Case:-0.84 (-2.05~-0.61) Non-case:-0.98 (-2.14~–0.40)	1.24(0.9,1.7)	21.3	Sex, country of birth, smoking, alcohol consumption, body mass index physical activity, education, and socioeconomic status
Shivappa5 et al.2017 [26]	Italy	Case: 690 Control: 665	Hospital based	Case: 25–80 Control: —	Case-control	Bladder	FFQ (95 items)	31	Case:-0.63±1.94 Co66ntrol:-0.93±2.00	1.97 (1.28, 3.03) Male:1.83 (1.14, 2.91) Female:5.73 (1.46, 22.44)	—	Age, sex, year of interview, study center, and total energy intake, education and tobacco smoking

Abbreviations: OR, odds ratio; HR, hazard ratio; FFQ, food frequency questionnaire; PC, prostate cancer; RCC, renal cell carcinoma; UCC, urothelial cell carcinoma; BMI, Body Mass Index; HRT, hormone replacement therapy.

**Table 2:** Quality assessment of all included studies.

First author	Publishing year	Selection	Comparability	Exposure	Total
Shivappa1 et al. [22]	2015	★★★	★	★★	6
Shivappa2 et al. [23]	2017	★★★	★★	★★	7
Shivappa3 et al. [28]	2016	★★★	★★	★★	7
Graffouillère et al. [17]	2016	★★★	★★	★★★	8
Vázquez-Salas et al. [19]	2016	★★★	★★	★★★	8
Shivappa4 et al. [25]	2015	★★★	★★	★★	7
Shivappa8 et al. [29]	2018	★★★	★★	★★	7
Shivappa9 et al. [30]	2018	★★★	★★	★★	7
Shivappa6 et al. [24]	2017	★★★	★★	★★	7
Shivappa7 et al. [27]	2017	★★★	★★	★★	7
Dugué et al. [18]	2016	★★★	★★	★★	7
Shivappa5 et al. [26]	2016	★★★	★★	★★	7

### DII score and UC risk

**Fig 1:**
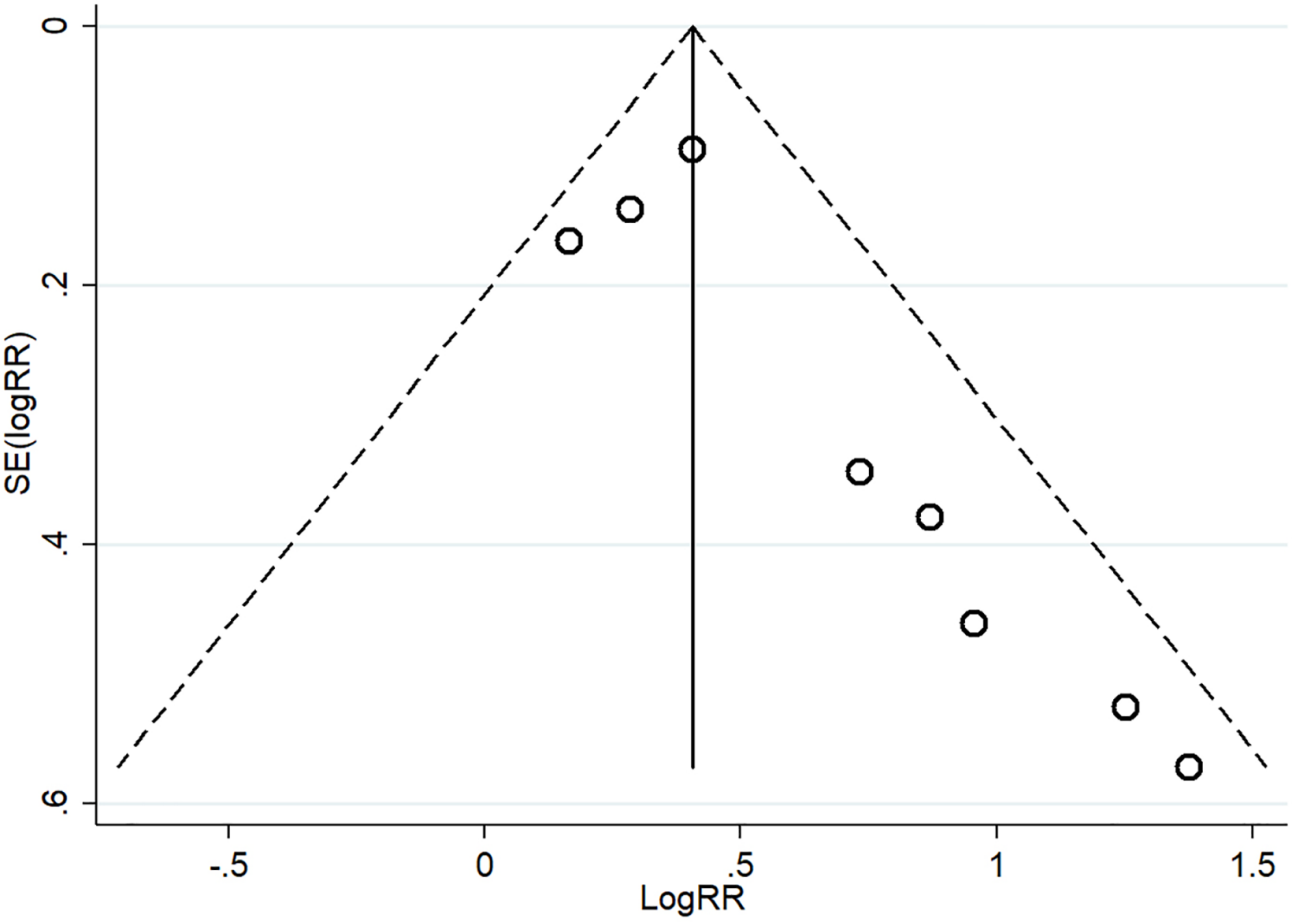
Funnel plot of the studies assessing the association between the DII score and prostate cancer risk.

**Fig 2:**
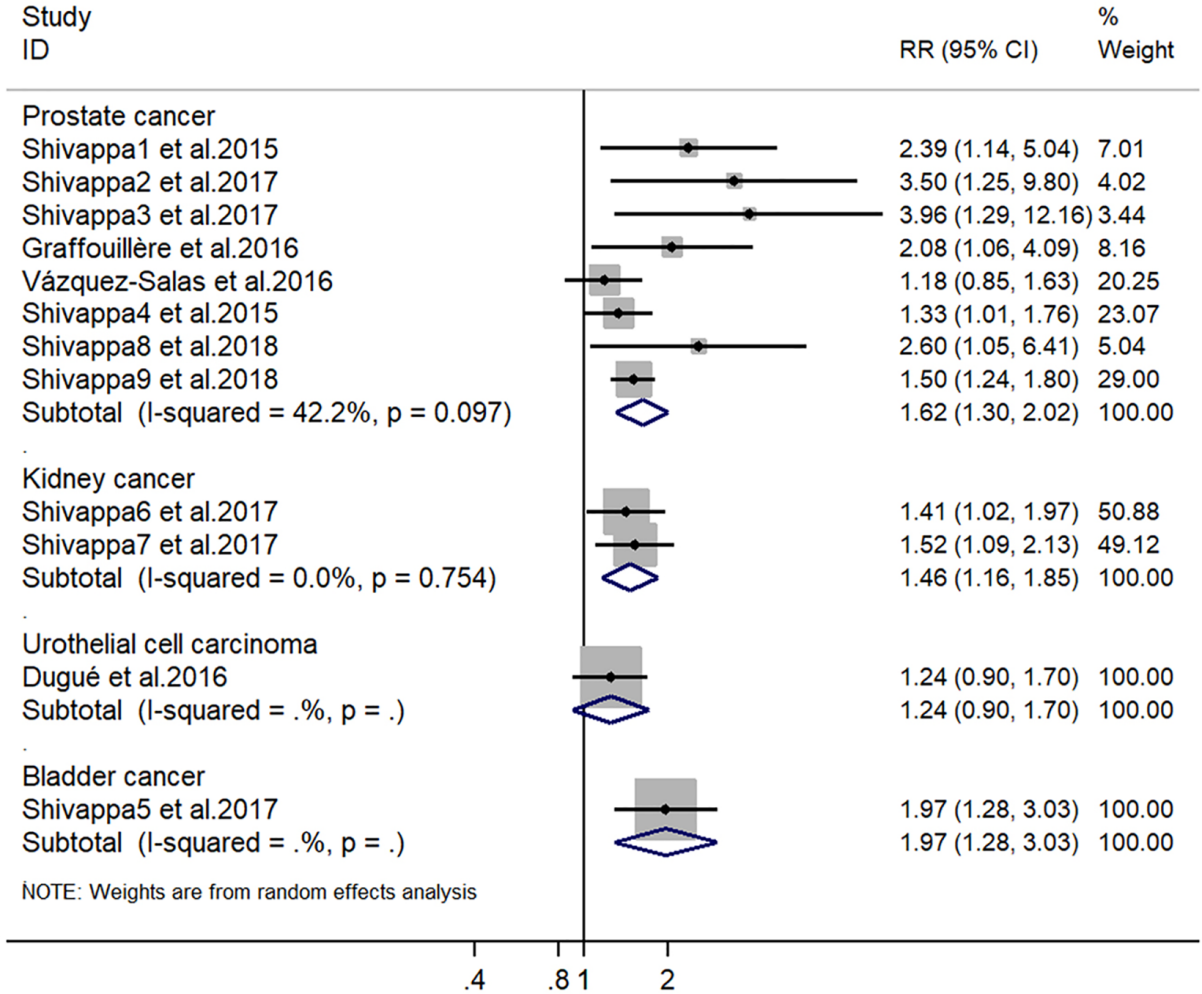
Forest plots showing RR with 95% CI of urologic cancer comparing the highest to the lowest dietary inflammatory index score.

**Fig 3:**
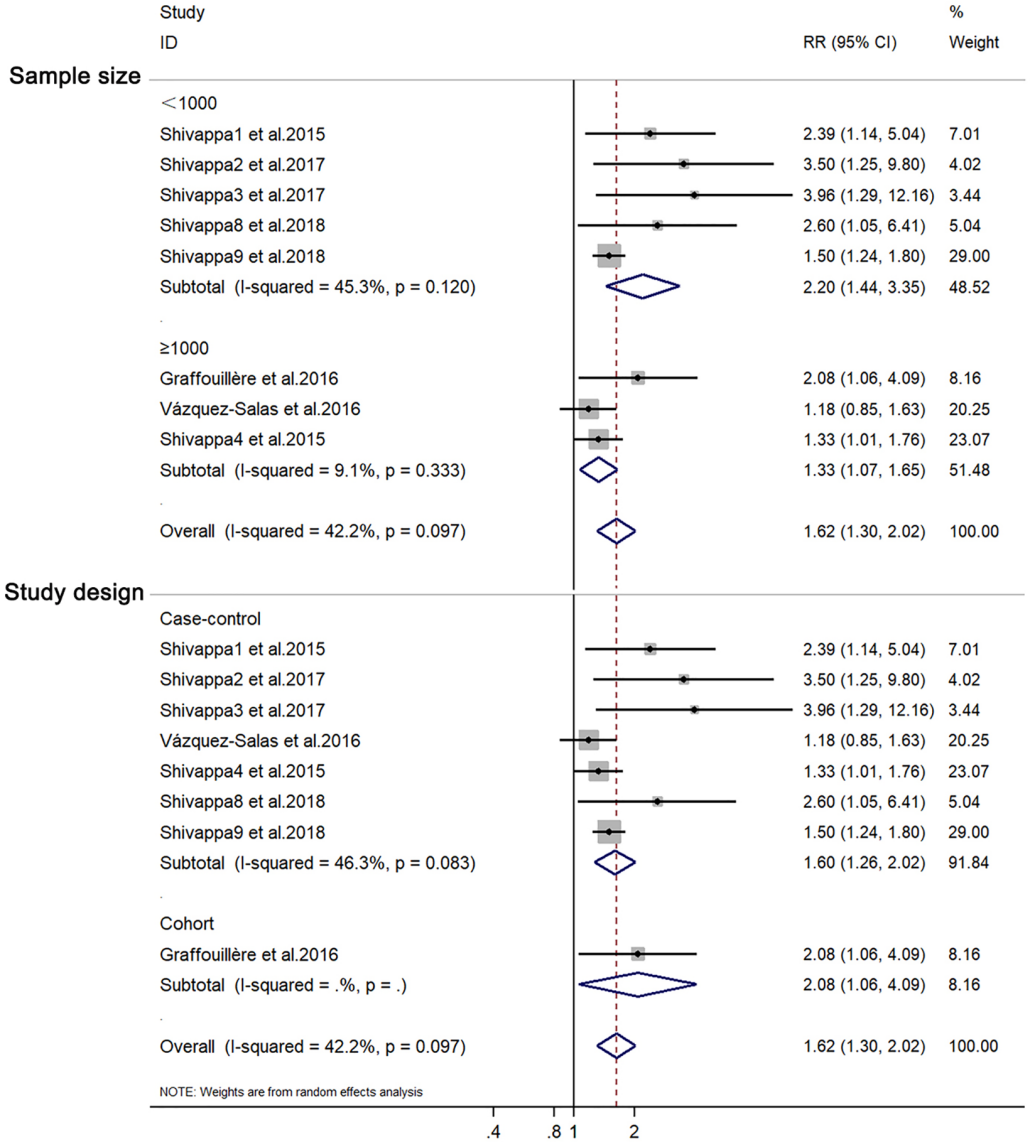
Forest plots showing RR with 95% CI of prostate cancer comparing the highest to the lowest dietary inflammatory index score.

Eight studies with 10,328 individuals in total evaluated the association of DII score with prostate cancer risk. Significant heterogeneity was found among the studies (I^2^ = 42.2%). Additionally, publication bias was observed from the Begg (P = 0.013) and Egger regression tests (P = 0.019), as well as the funnel plot (Fig 1). The pooled RR for the highest versus lowest DII score was 1.62 (95% CI: 1.30–2.02) (Fig 2). Subgroup analyses based on study design and sample size showed consistent results (Fig 3).

Two studies, including a total of 36,121 individuals, evaluated the association of DII score with kidney cancer risk. There was no significant heterogeneity among the studies (I^2^ = 0%). The pooled RR for the highest versus lowest DII score was 1.46 (95% CI: 1.16–1.85) (Fig 2).

One study including 1,355 participants evaluated the association of DII score with bladder cancer risk. The pooled RR for the highest versus lowest DII score was 1.97 (95% CI: 1.28–3.03). One cohort study with 37,442 participants evaluated the association of DII score with urothelial cell carcinoma risk. The pooled RR for the highest versus lowest DII score was 1.24 (95% CI: 0.90–1.70) (Fig 2).

### Sensitivity analyses and Publication bias

**Fig 4:**
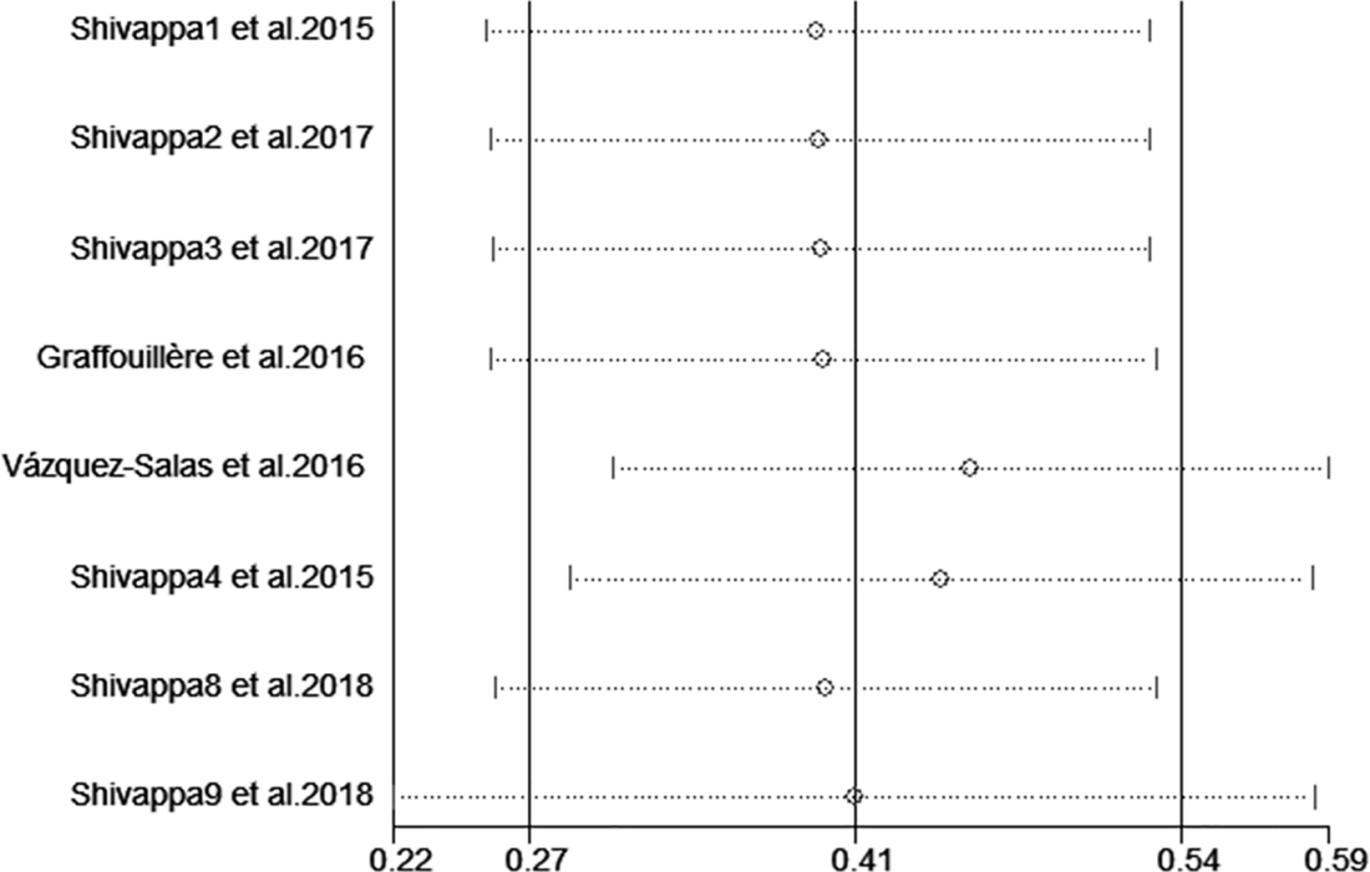
Sensitivity analysis diagram for each study used to assess the association between the DII score and prostate cancer risk.

Sensitivity analysis was performed for prostate cancer by omitting one study each time; the results showed that the overall pooled RRs were not influenced by any individual study (Fig 4), suggesting that the results of this meta-analysis are stable. Some publication bias was observed in the results according to Begg’s (P = 0.013) and Egger’s tests (P = 0.019) and funnel plots (Fig 1).

## Discussion

This is, to our knowledge, the first systematic review with a meta-analysis that evaluates the association between the inflammatory potential of diet and UC risk. Eleven studies with a total of 83,197 participants met the inclusion criteria and were finally included in the meta-analysis. The results showed that more pro-inflammatory diets, estimated by a higher DII score, are associated with an increased risk of prostate cancer, kidney cancer and bladder cancer.

The etiology of urologic cancers (including prostate, bladder, kidney cancers, and urothelial cell carcinoma) is complicated, and several risk factors are involved in their development and progression; in addition to environmental and genetic risk factors, lifestyle risk factors, such as dietary habits, also play important role in cancer development and progression [31–35]. There is growing evidence strongly supporting the involvement of inflammation in carcinogenesis [5, 36]. Diet represents a complex set of exposures that often interact, and cumulative effects may modify both inflammatory responses and health outcomes [22, 37]. Specific dietary components may decrease UC risk by influencing both acute and chronic inflammation.

In the present meta-analysis, a stronger association was detected between higher DII score and prostate cancer risks in the overall analysis. The pooled adjusted risk ratio (RR) for the highest DII score versus the lowest category was 1.62 (95% CI: 1.30–2.02). Subgroup analyses based on study design and sample size showed consistent results. Vázquez-Salas et al. reported that there is no evidence of an association between a pro-inflammatory diet and prostate cancer risk, in contrast to the conclusions of previous studies [19, 22, 25]. This difference among studies may be the result of small sample sizes, study design or population substructure, or other factors. For kidney cancer, the pooled adjusted RR of kidney cancer for the highest DII score versus the lowest category was 1.46 (95% CI: 1.16–1.85), which is consistent with that in previous studies [24, 27]. For bladder cancer and urothelial cell carcinoma, participants in the highest category of DII score were associated with an increased risk of bladder cancer (RR = 1.97, 95% CI: 1.28–3.03) compared with those in the lowest DII category [26]. However, a pro-inflammatory diet is not related to urothelial cell carcinoma risk (RR = 1.24, 95% CI: 0.90–1.70) [18]. Only two studies reported the relationship between the DII score and kidney cancer risk; one study for bladder cancer and one study for urothelial cell carcinoma were included in the present meta-analysis. The sample size was small; thus, studies with larger sample sizes are needed to further investigate the potential relationships of DII score with these cancer risks.

When interpreting the results of the current study, some limitations should be considered. First, only two studies reported the relationship between the DII score and kidney cancer risk; additionally, one study for bladder cancer and one study for urothelial cell carcinoma reported a relationship. The sample size of included published articles was small, so sufficient data was unavailable. Second, the DII score was calculated by self-report, inevitably leading to some recall bias. Third, substantial heterogeneity reporting on prostate cancer was observed among studies; this may a result of the different number of food parameters, geographical region and follow-up duration. Finally, some publication bias exists in the results which may due to the limited studies in the present meta-analysis.

## Conclusion

This study suggests that a pro-inflammatory diet is associated with an increased risk of prostate cancer, kidney cancer and bladder cancer. Nevertheless, more large-scale, well-designed studies are needed to investigate the findings, and future research is needed to investigate whether an anti-inflammatory dietary pattern could constitute a beneficial nutritional choice for the primary prevention of UC.

## Supporting information

S1 Checklist PRISMA checklist for this meta-analysis. (DOC)

S1 Fig Flowchart showing study selection. (DOC)
