# Supplementary material for: Meta-analysis of the association between the inflammatory potential of diet and urologic cancer risk
Source: PLoS One. 2018 Oct 1;13(10):e0204845. doi: 10.1371/journal.pone.0204845 (PMC6166946; doi:10.1371/journal.pone.0204845)
Supplement: S1 Fig — (DOC) [file pone.0204845.s002.doc]

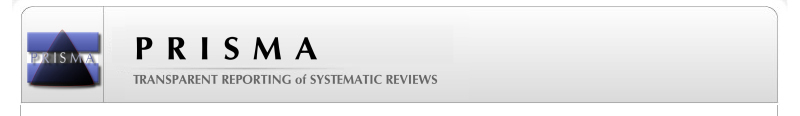
**PRISMA 2009 Flow Diagram**

**Screening**

**Included**

**Eligibility**

**Identification**

Records identified through database searching:
Pubmed (n=286), Embase (n=19), Web of science (n=550)

Additional records identified through other sources
(n=0)

Records after duplicates removed
(n=672)

Records screened
(n=672)

Records excluded:

Not relevant using title and abstract (n=651)

Full-text articles assessed for eligibility
(n=21)

Full-text articles excluded:

Review articles (n=3), not relevant (n=4), specific type cancer survival (n=1), other dietary score (n=1)

Studies included in qualitative synthesis
(n=12)

Studies included in quantitative synthesis (meta-analysis)
(n=12)
